# Supplementary material for: HIVprotI: an integrated web based platform for prediction and design of HIV proteins inhibitors
Source: J Cheminform. 2018 Mar 9;10:12. doi: 10.1186/s13321-018-0266-y (PMC5845081; doi:10.1186/s13321-018-0266-y)
Supplement: Supplementary file 2 — Additional file 2. Source code of HIVProtI web server. [file 13321_2018_266_MOESM2_ESM.zip › HIVprotI_Source-code/hivprotI/weka-3-6-6/doc/weka/attributeSelection/CostSensitiveAttributeEval.html]

CostSensitiveAttributeEval


---


|  |  |  |
| --- | --- | --- |
| **Overview** \| **Package** \| **Class** \| **Tree** \| **Deprecated** \| **Index** \| **Help** \| **Weka's home** | |  |
| **PREV CLASS**   **NEXT CLASS** | **FRAMES**    **NO FRAMES**     **All Classes** |  |
| SUMMARY: NESTED \| FIELD \| CONSTR \| METHOD | DETAIL: FIELD \| CONSTR \| METHOD |  |


---


## weka.attributeSelection Class CostSensitiveAttributeEval

```
java.lang.Object
  weka.attributeSelection.ASEvaluation
      weka.attributeSelection.CostSensitiveASEvaluation
          weka.attributeSelection.CostSensitiveAttributeEval
```

**All Implemented Interfaces:**: java.io.Serializable, AttributeEvaluator, CapabilitiesHandler, OptionHandler, RevisionHandler

---

``` public class CostSensitiveAttributeEval extends CostSensitiveASEvaluation implements java.io.Serializable, AttributeEvaluator, OptionHandler ```

A meta subset evaluator that makes its base subset evaluator cost-sensitive.


Valid options are:

```
 -C <cost file name>
  File name of a cost matrix to use. If this is not supplied,
  a cost matrix will be loaded on demand. The name of the
  on-demand file is the relation name of the training data
  plus ".cost", and the path to the on-demand file is
  specified with the -N option.
```

```
 -N <directory>
  Name of a directory to search for cost files when loading
  costs on demand (default current directory).
```

```
 -cost-matrix <matrix>
  The cost matrix in Matlab single line format.
```

```
 -S <integer>
  The seed to use for random number generation.
```

```
 -W
  Full name of base evaluator.
  (default: weka.attributeSelection.ReliefFAttributeEval)
```

```
 Options specific to evaluator weka.attributeSelection.ReliefFAttributeEval:
```

```
 -M <num instances>
  Specify the number of instances to
  sample when estimating attributes.
  If not specified, then all instances
  will be used.
```

```
 -D <seed>
  Seed for randomly sampling instances.
  (Default = 1)
```

```
 -K <number of neighbours>
  Number of nearest neighbours (k) used
  to estimate attribute relevances
  (Default = 10).
```

```
 -W
  Weight nearest neighbours by distance
```

```
 -A <num>
  Specify sigma value (used in an exp
  function to control how quickly
  weights for more distant instances
  decrease. Use in conjunction with -W.
  Sensible value=1/5 to 1/10 of the
  number of nearest neighbours.
  (Default = 2)
```

**Version:**
:   $Revision: 5562 $

**Author:**
:   Mark Hall (mhall{[at]}pentaho{[dot]}com)

**See Also:**: Serialized Form

---

**Field Summary**

**Fields inherited from class weka.attributeSelection.CostSensitiveASEvaluation**

`MATRIX_ON_DEMAND, MATRIX_SUPPLIED, TAGS_MATRIX_SOURCE`


**Constructor Summary**

`CostSensitiveAttributeEval()`
  
          Default constructor.


| **Method Summary** | |
| --- | --- |
| `java.lang.String` | `defaultEvaluatorString()`             Return the name of the default evaluator. |
| `double` | `evaluateAttribute(int attribute)`             Evaluates an individual attribute. |
| `java.lang.String` | `getRevision()`             Returns the revision string. |
| `static void` | `main(java.lang.String[] args)`             Main method for testing this class. |
| `void` | `setEvaluator(ASEvaluation newEvaluator)`             Set the base evaluator. |

**Methods inherited from class weka.attributeSelection.CostSensitiveASEvaluation**

`buildEvaluator, costMatrixSourceTipText, costMatrixTipText, evaluatorTipText, getCapabilities, getCostMatrix, getCostMatrixSource, getEvaluator, getOnDemandDirectory, getOptions, getSeed, globalInfo, listOptions, onDemandDirectoryTipText, postProcess, seedTipText, setCostMatrix, setCostMatrixSource, setOnDemandDirectory, setOptions, setSeed, toString`

**Methods inherited from class weka.attributeSelection.ASEvaluation**

`forName, makeCopies`

**Methods inherited from class java.lang.Object**

`equals, getClass, hashCode, notify, notifyAll, wait, wait, wait`

**Methods inherited from interface weka.core.OptionHandler**

`getOptions, listOptions, setOptions`

**Constructor Detail**

### CostSensitiveAttributeEval

```
public CostSensitiveAttributeEval()
```

:   Default constructor.


**Method Detail**

### defaultEvaluatorString

```
public java.lang.String defaultEvaluatorString()
```

:   Return the name of the default evaluator.

    :   **Overrides:**: `defaultEvaluatorString` in class `CostSensitiveASEvaluation`
    :   **Returns:**: the name of the default evaluator

---


### setEvaluator

```
public void setEvaluator(ASEvaluation newEvaluator)
                  throws java.lang.IllegalArgumentException
```

:   Set the base evaluator.

    :   **Overrides:**: `setEvaluator` in class `CostSensitiveASEvaluation`
    :   **Parameters:**: `newEvaluator` - the evaluator to use. **Throws:**: `java.lang.IllegalArgumentException` - if the evaluator is not an instance of AttributeEvaluator

---


### evaluateAttribute

```
public double evaluateAttribute(int attribute)
                         throws java.lang.Exception
```

:   Evaluates an individual attribute. Delegates the actual evaluation to the
    base attribute evaluator.

    :   **Specified by:**: `evaluateAttribute` in interface `AttributeEvaluator`
    :   **Parameters:**: `attribute` - the index of the attribute to be evaluated **Returns:**: the "merit" of the attribute **Throws:**: `java.lang.Exception` - if the attribute could not be evaluated

---


### getRevision

```
public java.lang.String getRevision()
```

:   Returns the revision string.

    :   **Specified by:**: `getRevision` in interface `RevisionHandler` **Overrides:**: `getRevision` in class `CostSensitiveASEvaluation`
    :   **Returns:**: the revision

---


### main

```
public static void main(java.lang.String[] args)
```

:   Main method for testing this class.

    :   **Parameters:**: `args` - the options


---


|  |  |  |
| --- | --- | --- |
| **Overview** \| **Package** \| **Class** \| **Tree** \| **Deprecated** \| **Index** \| **Help** \| **Weka's home** | |  |
| **PREV CLASS**   **NEXT CLASS** | **FRAMES**    **NO FRAMES**     **All Classes** |  |
| SUMMARY: NESTED \| FIELD \| CONSTR \| METHOD | DETAIL: FIELD \| CONSTR \| METHOD |  |


---
